# Fitness Landscape of the Fission Yeast Genome

**DOI:** 10.1093/molbev/msz113

**Published:** 2019-05-11

**Authors:** Leanne Grech, Daniel C Jeffares, Christoph Y Sadée, María Rodríguez-López, Danny A Bitton, Mimoza Hoti, Carolina Biagosch, Dimitra Aravani, Maarten Speekenbrink, Christopher J R Illingworth, Philipp H Schiffer, Alison L Pidoux, Pin Tong, Victor A Tallada, Robin Allshire, Henry L Levin, Jürg Bähler

**Affiliations:** 1 Department of Genetics, Evolution and Environment, University College London, London, United Kingdom; 2 Department of Biology and York Biomedical Research Institute, University of York, United Kingdom; 3 Experimental Psychology, University College London, London, United Kingdom; 4 Department of Genetics, University of Cambridge, Cambridge, United Kingdom; 5 Wellcome Trust Centre for Cell Biology, University of Edinburgh, Edinburgh, United Kingdom; 6 Centro Andaluz de Biología del Desarrollo, Universidad Pablo de Olavide/Consejo Superior de Investigaciones Científicas, Seville, Spain; 7 Division of Molecular and Cellular Biology, Eunice Kennedy Shriver National Institute of Child Health and Human Development, National Institutes of Health, Bethesda, MD, USA; 8 UCL Genetics Institute, University College London, London, United Kingdom

**Keywords:** *Schizosaccharomyces pombe*, transposon mutagenesis, gene function, cellular fitness, noncoding genome, Tn-Seq

## Abstract

The relationship between DNA sequence, biochemical function, and molecular evolution is relatively well-described for protein-coding regions of genomes, but far less clear in noncoding regions, particularly, in eukaryote genomes. In part, this is because we lack a complete description of the essential noncoding elements in a eukaryote genome. To contribute to this challenge, we used saturating transposon mutagenesis to interrogate the *Schizosaccharomyces pombe* genome. We generated 31 million transposon insertions, a theoretical coverage of 2.4 insertions per genomic site. We applied a five-state hidden Markov model (HMM) to distinguish insertion-depleted regions from insertion biases. Both raw insertion-density and HMM-defined fitness estimates showed significant quantitative relationships to gene knockout fitness, genetic diversity, divergence, and expected functional regions based on transcription and gene annotations. Through several analyses, we conclude that transposon insertions produced fitness effects in 66–90% of the genome, including substantial portions of the noncoding regions. Based on the HMM, we estimate that 10% of the insertion depleted sites in the genome showed no signal of conservation between species and were weakly transcribed, demonstrating limitations of comparative genomics and transcriptomics to detect functional units. In this species, 3′- and 5′-untranslated regions were the most prominent insertion-depleted regions that were not represented in measures of constraint from comparative genomics. We conclude that the combination of transposon mutagenesis, evolutionary, and biochemical data can provide new insights into the relationship between genome function and molecular evolution.

## Introduction

A goal of genetics is to understand what sequence elements within genomes specify cellular and organismal function. The highly transcribed protein-coding regions of eukaryote genomes are routinely detected within genomes and are well studied. The numerous noncoding elements, on the other hand, are more challenging to detect, profile, and functionally describe. While biochemical assays of genome activity can indicate functional units, inferring function based *solely* on biochemical activity, for example, the ENCODE project’s definition of functional DNA (ENCODE Project Consortium et al. 2012), is inconsistent with evolutionary analyses that show no signal of conservation for substantial proportions of larger eukaryotic genomes (Doolittle 2013; Graur et al. 2013).

In theory, functionally important elements could be detected by their conservation between lineages relative to neutral elements. However, such analyses suffer from the paradox that more divergent species allow more sensitive detection of small functional elements, but there will be fewer shared functional regions (Stone et al. 2005). Similarly, patterns of diversity detect evolutionarily constrained regions within a species (Fawcett et al. 2014; Jeffares et al. 2015; Yu et al. 2015). However, these analyses are limited to summaries of annotation types, rather than defining particular conserved elements, because segregating genetic variants are generally too sparse within specific genes to estimate the fitness effects of mutations accurately. Additionally, various factors can affect segregating variants and/or allele frequencies at any particular genomic locus, including recombination rate (Campos et al. 2014) and recent events of selection which purge diversity in surrounding areas (Smith and Haigh 1974; Cheeseman et al. 2012). For these reasons, neither diversity nor divergence analyses have sufficient power to describe functional constraint at gene or subgenic resolution. In contrast, high-density transposon-insertion libraries generated from independent repeats can precisely define functional elements and have provided estimators of gene-knockout fitness in bacterial genomes (van Opijnen et al. 2009; Zhang et al. 2012; DeJesus and Ioerger 2013; Chao et al. 2016; Price et al. 2018). A limitation of transposon mutagenesis screens is that some regions will have functional roles that are specific to particular environments, developmental stages, or genetic backgrounds (Price et al. 2018).

To define functional elements in a eukaryote genome, we generated multiple dense insertion libraries in fission yeast (*Schizosaccharomyces pombe*), using the *Hermes* cut and paste transposon system (Park et al. 2009). We analyzed these data with respect to genome annotation, genetic diversity, divergence, and transcriptional output. We then developed a hidden Markov model (HMM) in an effort to account for biases in insertion frequency and smooth the stochastic insertion profiles into meaningful measures of insertion-fitness profiles that span multiple continuous genome positions. Both the raw insertion density metric and the HMM states showed significant relationships to independent predictors of functional elements, including evolutionary constraint, genetic diversity, annotation boundaries, and transcript levels. Therefore, these data provide a rich resource for further study of genic and nongenic functional elements.

## Results

### Generation of Dense *Hermes* Insertion Libraries in Fission Yeast

We generated nine *Hermes* insertion libraries using modifications of previously published methods (Evertts et al. 2007; Park et al. 2009; Guo et al. 2013). Insertions were generated in cultures undergoing rapid mitotic proliferation, serially diluted for ∼25 cell divisions (supplementary fig. 1, Supplementary Material online). Insertion sites were identified using a custom *Hermes*-end primed sequencing strategy to produce paired-end reads (supplementary fig. 2, Supplementary Material online). This approach included the attachment of a 10-nucleotide (nt) unique molecular identifier (UMI) to each sequenced DNA molecule, which enabled us to remove PCR-generated duplicates of *Hermes*-containing DNA molecules and thus count the number of insertions per position. These counts represent either multiple independent insertions at a genomic location (in different cells within a library), or the result of a single insertion event that has been propagated by cell division.

The libraries contained an average of 1.6 million genomic insertions (supplementary table 1, Supplementary Material online). Collectively, our libraries contained 31 million insertions at 930,000 unique sites, an average insertion density of 1 insertion site per 13 nt of the genome.

### Insertion Density is Consistent with Expectations of Functional Constraint

Based on previous transposon analyses in bacteria and yeasts, we expected that more important regions would tolerate fewer insertions (Guo et al. 2013; Chao et al. 2016; Michel et al. 2017). Initial analysis showed that both insertion density (unique insertion positions/site) and average insertion count (insertion instances per site) were significantly lower in essential genes compared with nonessential genes and higher in nongenic regions (supplementary fig. 3, Supplementary Material online). This result suggested that insertions reflect the relative functional importance of these annotated elements.

Notably, the mitochondrial genome also featured high insertion density, but with little difference between coding and noncoding regions (supplementary fig. 4, Supplementary Material online). This result likely reflects that any given transposon insertion among multiple mitochondrial genomes will have little or no consequence for the cell. Nevertheless, this finding shows that *Hermes* transposition can readily occur in mitochondria.

**Fig. 1.**
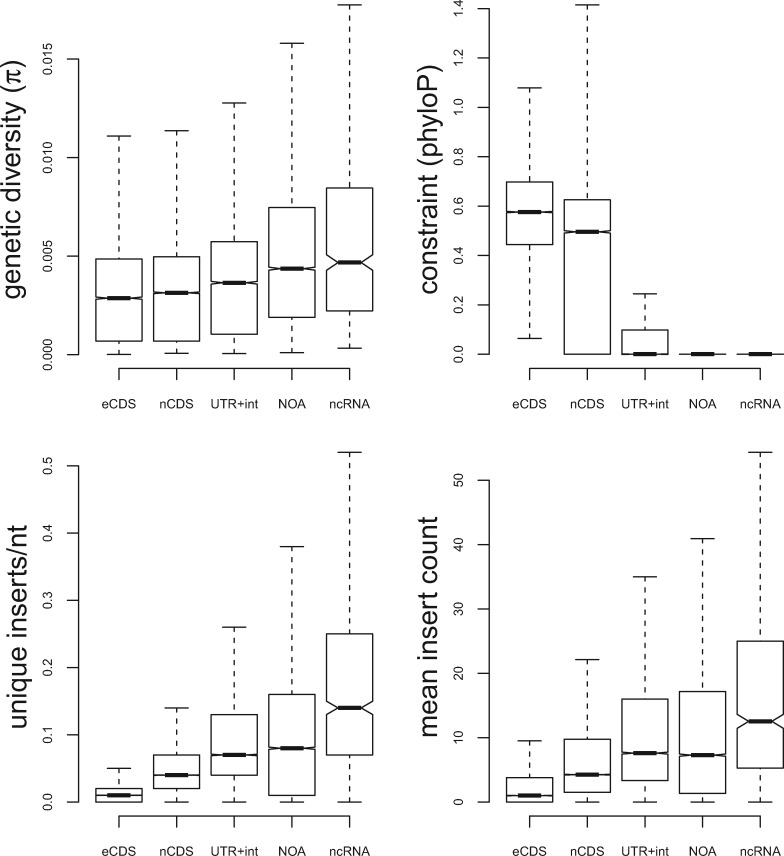
*Hermes* insertion data recapitulate signals of evolutionary constraint. For protein-coding regions of essential genes (eCDS), protein-coding regions of nonessential genes (nCDS), 5′/3′-UTRs and introns (UTR+int), regions of the genome without any annotation (NOA) and noncoding RNAs (ncRNAs) we show: (*A*) the genetic diversity from 57 strains of *Schizosaccharomyces pombe* (Jeffares et al. 2015), measured in 100-nt windows, and (*B*) the phyloP measure of constraint (Gagliano et al. 2014) between four *Schizosaccharomyces* species (mean phyloP score, over 100-nt windows). Similarly, for pooled proliferation *Hermes* data, we show: (*C*) the number of unique insertions/nt, and (*D*) the mean insertion counts (calculated including sites without insertions as zero counts). All these plots exclude outliers.

To systematically examine the relationship between genomic regions and insertions, we compared our *Hermes* insertion data with genetic diversity (π), within *S.pombe* strains and divergence between *Schizosaccharomyces* species. Based on these evolutionary measures of functional constraint, we divided the genome into five annotation classes: coding regions of essential genes, coding regions of nonessential genes, 5′/3′-untranslated regions (UTRs) and introns, genomic regions with no annotation (generally intergenic regions), and noncoding RNAs. The relative levels of genetic diversity and divergence consistently showed that essential coding regions were subject to higher constraint than nonessential coding regions, followed by UTRs/introns, with unannotated regions being the least constrained. *Hermes* insertion density (unique insertions/nt) and mean insertion count were consistent with this ranking (fig. 1). These findings suggest that *Hermes* insertion density has a meaningful quantitative relationship to evolutionary constraint, even though insertions were generated in only one culture condition.

### Application of an HMM to account for Insertion Biases

Previous analyses have shown that the *Hermes* transposon insertions are biased toward nucleosome-free DNA and that they preferentially occur in DNA with a degenerate sequence motif (TNNNNA) (Gangadharan et al. 2010; Guo et al. 2013). We sought to develop a prediction of the fitness consequences of transposon insertions at a fine-scale resolution correcting for such bias. This prediction should also reflect that neighboring nucleotides in a genome do not function independently but as “functional” units (e.g., exons, introns, UTRs). We developed an HMM to correct for these insertion biases and smooth the signal from stochastic insertions into contiguous functional units. In this model, the observed data are the insertion counts and the “hidden” state is the degree of biological importance. Regions with greater importance are expected to have fewer insertions.

Our model utilized measurements of nucleosome density and sequence composition. Genome-wide profiles of nucleosome density were obtained from proliferating cells (Atkinson et al. 2018). Next, the sequence composition of previously recorded in vitro insertion sites (Guo et al. 2013) were evaluated to find a degenerate insertion motif. We then constructed a sequence composition measure, termed insertion motif similarity score (IMSS), which describes the similarity of each position in the genome to this motif. Data from these two measurements were used to construct generalized linear models describing the relationship between insertion density, nucleosome density, and IMSS (supplementary fig. 5, Supplementary Material online).

Our HMM divided the genome into five states, from state 1 (S1), indicating the sites at which transposon insertion had the greatest negative functional consequences, to state 5 (S5), indicating sites at which insertion had the least negative (or potentially positive) functional consequences. This number of states was obtained from initial trials with the model, detailed below. Annotated regions of the genome were used to train the model. The first state, S1, was trained on coding regions of essential genes (whose knockouts are inviable), S2 was trained on coding regions of nonessential genes, S3 on regions that may have some importance but weaker signals (introns and UTRs), S4 on unannotated intergenic regions that show high genetic diversity (Jeffares et al. 2015), where mutations or insertions may be neutral, and S5 on the top-10% insertion-dense sites to allow for the possibility that insertions in some positions enhance cell survival.

The model was fitted to the data by maximum likelihood, using the EM algorithm. The Viterbi algorithm was then used to determine the most likely state (S1–S5) for each genomic position given the nucleosome density, IMSS, and insertion counts. Model fitting did not explicitly include annotations (see Materials and Methods for details on HMM). HMM states were highly consistent between independent HMM model fitting runs (see Materials and Methods). Insertion data, HMM states, nucleosome density, and conservation measures are available in a dedicated genome browser http://bahlerweb.cs.ucl.ac.uk/bioda (Firefox browser compatible; Accessed on May 10, 2019) and in the fission yeast model organism database PomBase (www.pombase.org). These tools allow users to check functional information for regions of interest, including fine-scale structure–function relationships within specific genes and putative regulatory regions.

### Validity and Limitations of the Model

**Fig. 2.**
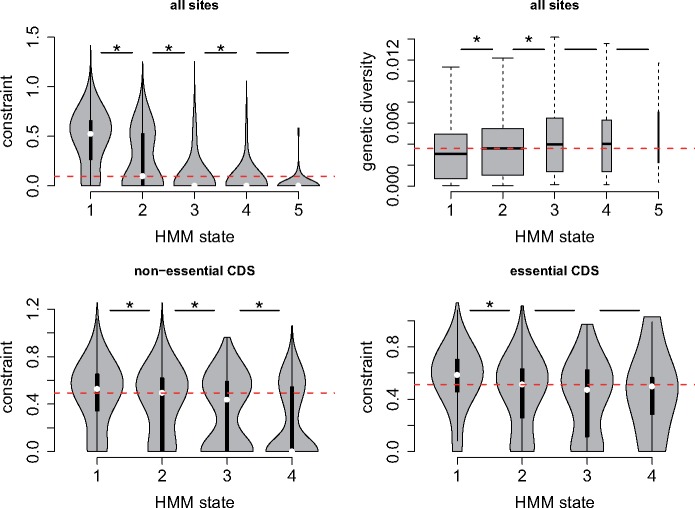
HMM states are indicative of conserved, functionally significant regions. (*A*) For all 100-nt windows in the genome, we show constraint distributions for mean HMM states S1–S5. Comparisons of S1 versus S2, S2 versus S3, and S3 versus S4 were significantly different (Wilcoxon rank sum tests, all *P* < 10^−16^), indicated by asterisks. (*B*) Similarly, we show diversity for windows with mean HMM states S1–S5. Bar widths are proportional to the number of windows, with outliers being excluded. Comparisons of S1 versus S2 and S2 versus S3 were significantly different (Wilcoxon rank sum tests, *P* < 10^−16^ and *P* = 4×10^−13^). (*C*) For windows within nonessential protein-coding regions, constraint distributions S1 versus S2, S2 versus S3 and S3 versus S4 were significantly different (Wilcoxon rank sum tests, *P* < 10^−16^, *P* = 1×10^−8^, *P* = 8×10^−3^). (*D*) For 100-nt windows within essential protein-coding regions, only constraint distributions S1 versus S2 were significantly different (Wilcoxon rank sum tests, *P* < 10^−16^), but <1% of windows were in categories with mean HMM states >2. In all plots, horizontal red lines show median values for HMM state 2 category windows, and black horizontal bars indicate categories that were significantly different by Wilcoxon rank sum tests. All plots show differences in metrics over 100-nt windows. Constraint was calculated as the mean phyloP measure (see Materials and Methods) of conservation for sites within the window, and genetic diversity is average pairwise difference (π) from 57 nonclonal *Schizosaccharomyces pombe* strains (Jeffares et al. 2015). Mean HMM states utilized only windows where 95% of the positions were read-mappable, to preclude low-mapping windows masquerading as insertion-depleted windows. HMM state categories 1 and 2 were defined as windows where the mean HMM states were exactly 1 or 2 (which was frequent). HMM state categories 3–5 were defined as windows with mean HMMs within 0.5 of this range (e.g., state 3 category >2.5 and <3.5).

To evaluate the validity of the HMM, we first examined the relationship between HMM states, rates of divergence in *Schizosaccharomyces* species, and diversity within *S. pombe.* If lower HMM states (fewer insertions) were indicative of more functionally important regions, we would expect them to show lower divergence and less genetic diversity due to increased constraint. To examine this expectation, we divided the genome into 126,311 windows of 100 nt, and calculated insertion density (unique insertion sites/100 nt), mean HMM state, mean constraint (using the phyloP algorithm), and average pairwise diversity (π) within *S. pombe* strains. We found that lower HMM states were subject to significantly higher constraint (fig. 2*A*). Similarly, lower HMM states showed significantly lower genetic diversity, consistent with greater purifying selection within *S. pombe* (fig. 2*B*). This result is consistent with the notion that HMM states S1–S3 have biological relevance, and that S1 and S2 represent functionally important regions.

This finding was not surprising, given that HMM states are strongly correlated with insertion density (Pearson *r* = 0.78), and we had already established that coding regions contained lower insertion densities (fig. 1). It is possible that this genome-wide pattern could reflect merely differences between coding and noncoding regions, which have different constraints but also differ in GC content, nucleosome densities, and other features which might influence transposon insertions and the usefulness of the HMM.

To examine whether the HMM could differentiate sites that were more/less important within one annotation-class, we examined HMM states within the protein-coding regions of nonessential genes. We found a statistically significant relationship between HMM states and constraint during evolution (fig. 2*C*). The relationship was present, but weaker, within coding regions of essential genes (fig. 2*D*). Hence, HMM states are indicative of conserved, functionally relevant regions genome-wide, but only weakly effective at predicting functional elements within protein-coding genes.

HMM has the potential advantage that it could enable discrete segmentation of genome windows to define boundaries of functional elements, unlike raw insertion density. To examine whether this model-based segmentation was biologically meaningful, we measured the relationship of the model to previously annotated elements, such as exons, introns, 5′- and 3′-UTRs, and noncoding RNAs. We defined 256,815 genomic regions that feature a continuous run of one HMM state (“HMM-defined elements,” HDEs). All S4 or S5 HDEs were <100 nt and mostly intergenic, indicating that only short regions in this genome can tolerate insertions without affecting fitness.

**Fig. 3.**
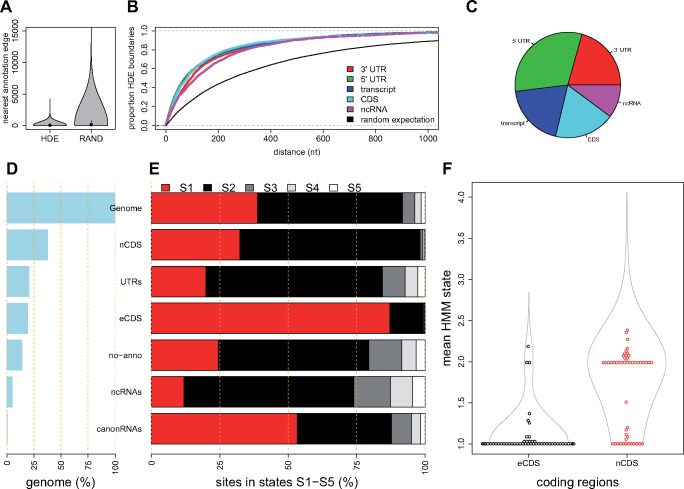
Functional Landscape by Annotation Type. Parts (*A*–*C*) show that the boundaries of HMM-Defined Elements (HDEs) are aligned to, or close to, the boundaries of existing annotations, as defined in legend at top right. The random expectation is derived from the same number of elements of the same lengths, placed at random on the genome. (*A*) HDEs have a smaller distance to the nearest annotation than random expectation. (*B*) For all HDE edges we show a cumulative density plot of nearest annotation type, including 5′/3′-UTRs, transcripts (transcription start/stop positions), coding sequences (amino-acid encoding regions, CDS), noncoding RNAs (ncRNAs), with lines colored according to the legend at right. (*C*) HDEs fell closest to a variety of annotations. The pie chart shows the proportions of nearest annotations, indicating a bias toward defining 5′-UTR edges. There were subtle differences between states S1, S2, and S3 in this respect (not shown). The HMM defined five states based on *Hermes* transposon insertions. State 1 (S1) refers to the most important regions, with the least insertions, and state 5 (S5) with the highest density of insertions. (*D*) Percentage of *Schizosaccharomyces pombe* genome covered by various annotation types: entire genome (100%), essential protein-coding regions (eCDS), protein-coding nonessential regions (nCDS), canonical noncoding RNAs (snRNAs, snpRNAs, tRNAs, rRNAs, canonRNAs), 5′/3′-UTRs (UTRs), noncoding RNAs (ncRNAs), and unannotated regions (no-anno). (*E*) Proportions of each annotation type in the five states: S1 (red), S2 (black), S3 (dark gray), S4 (light gray), and S5 (white). (*F*) Mean HMM states for essential (eCDS) and nonessential (nCDS) coding regions. Representative 50 points are shown for each type to indicate that most essential coding regions have mean state ∼1 (85% mean state <1.2).

We excluded these S4/S5 HDEs from further analysis, leaving 10,015 HDEs with a median length of 618 nt, which accounted for 90% of the mappable genome. HDE edges were closer to edges of existing annotations than expected by chance (Wilcoxon rank sum test, *P* < 10^−16^, fig. 3*A* and *B*). This result is consistent with these HMM-defined regions representing boundaries of various biologically relevant elements (including transcriptional units, spliced exons, or protein-coding regions).

Collectively, these findings are consistent with the HMM states S1–S3 showing a meaningful relationship to evolutionary constraint and boundaries of HMM-elements being aligned to exiting annotations. This analysis is consistent with states S1 and S2 being enriched for conserved, functionally important regions. A limitation of the HMM is that states S4 and S5 were not significantly different in any biological measure, so their meaning, if any, thus remains unclear.

### Genome-Wide Fitness Consequences of Insertions

Our analysis showed that 100-nt windows with HMM states S1/S2 are significantly more constrained within *Schizosaccharomyces* species, and feature less genetic diversity within *S. pombe* than regions with HMM states S3–S5 (fig. 2). As 91% of the genome was assigned to states S1/S2, a simplistic conclusion would be that transposon insertions have negative fitness consequences over 91% of the genome. Broadly, model states are consistent with known molecular biology. For example, 87% of the coding regions of essential genes were assigned to S1, compared with 32% of nonessential protein-coding regions, and smaller proportions of 5′- and 3′-UTRs, which account for a large proportion of the noncoding genome of *S. pombe* (fig. 3).

This modeling of insertions would suggest that most of the noncoding genome in this species contains functional elements. For example, the HMM assigned 82% nonprotein-coding regions to S1 or S2, indicating that they were strongly insertion-depleted relative to genome-wide expectations. UTRs, ncRNAs, and unannotated regions were each insertion-depleted to some extent (fig. 3*E*). This measure far exceeds the proportion that would be defined as important with the limited comparative genomics data available. For example, 24% of regions with no functional annotation are strongly insertion-depleted (S1), yet these regions show little conservation between *Schizosaccharomyces* species (fig. 1).

If we use insertions/site as an alternative model-free metric, noncoding regions also appear to contain functional units, though details differ. For example, if we assume that 95% of the coding regions of essential genes will be insertion-depleted due to the fitness consequences of insertions, we can establish a threshold for the insertion density of functional sites, as the 95th percentile of insertion density in essential coding regions (9 insertions/100 nt). We find that 66% of the genome has fewer insertions than this threshold, including 41% of the noncoding genome. This 41% of the noncoding genome is significantly more conserved than the remainder of the noncoding genome, consistent with this simple method being sufficient to detect functionally important regions (mean phyloP low-insertion noncoding regions 0.059, high-insertion noncoding regions 0.037, Wilcoxon rank sum test *P* < 10^−16^). Similarly, 50% of UTRs and 48% of regions with no annotations are below this threshold; in both cases, low-insertion regions are significantly more conserved than high-insertion regions with the same annotation (both Wilcoxon tests *P* < 10^−15^). These results indicate that insertion densities can predict sites that are likely functional, independently of, and consistently with, the HMM.

### Transposon Insertion Metrics Correlate with Gene Knockout Fitness

To examine whether raw insertion densities and/or the HMM contained information about the relative fitness cost of gene disruption, we calculated the mean HMM state and unique insertion sites/nt for each protein-coding gene (supplementary table 2, Supplementary Material online). As expected, essential coding genes had much lower metrics (fig. 1). To examine further whether these insertion metrics contained quantitative information about gene disruption fitness, we compared these measures to the colony sizes of viable knockout mutants on solid media (Malecki and Bähler 2016; Malecki et al. 2016). This orthogonal measure of gene disruption fitness alteration uses solid media (insertion metrics use liquid media), a more direct fitness measure, and different methods to interfere with gene function (disruption vs. deletion). We found that both metrics were positively correlated with the colony size of knockout mutants (inserts/nt, Pearson *r *=* *0.28, Spearman *r* = 0.30, mean HMM state Pearson *r* = 0.34, Spearman 0.25, all *P* < 10^−16^). Similarly, insertion metrics were correlated with constraint (mean phyloP vs. both inserts/nt and vs. mean HMM state, Pearson *r* = 0.30, *P* < 10^−16^). Other measures of knockout fitness collected from Bar-seq experiments of pooled mutants in liquid media (Kim et al. 2010; Sideri et al. 2014) were less correlated with both constraint and *Hermes* transposon insertion metrics (all correlations <0.1), suggesting that these laboratory fitness measures are limited in their power to predict long-term evolutionary constraints. In summary, although correlations are modest, these analyses indicate that the insertion metrics recover biologically meaningful fitness measures that add value beyond the binary classification of essential versus nonessential genes obtained from whole-gene disruptions.

### Characterizing HMM-Defined Functional Elements

**Fig. 4.**
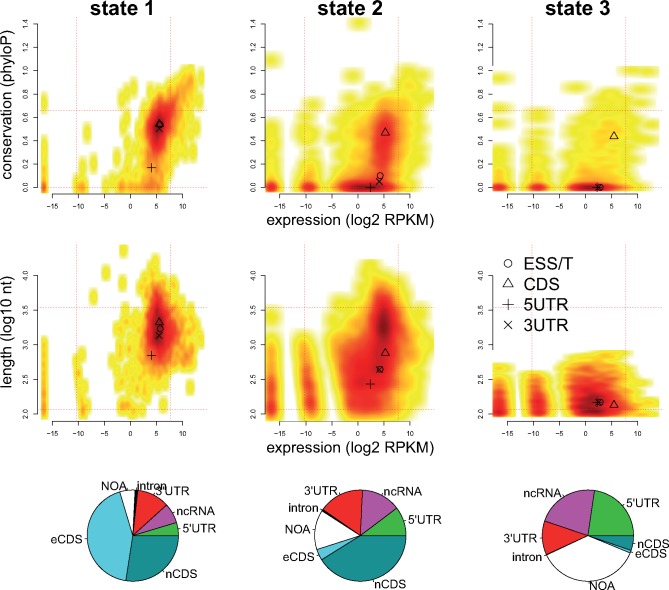
HMM-defined elements describe functional genomic outputs. Density plots describe various characteristics of HDEs, from left showing S1, S2, and S3 HDEs. Conservation (*y* axis, top row) levels are mean phyloP measures from four *Schizosaccharomyces* species. HDE lengths (*y* axis, middle row) are shown on a log_10_ scale. Expression levels (*x* axes) are RNA-Seq RPKMs from proliferating cells. Dashed horizontal and vertical lines show the 5th and 95th percentiles of conservation, expression levels or lengths. The positions of symbols (circle and triangle) indicate the median positions within each state for essential transcripts (ESS/T), coding regions (CDS), and 5′/3′-UTRs. For example, the few conserved S3 sites are coding regions. The bottom row shows the proportions of bases in S1, S2, and S3 HDEs that are annotated as introns, 3′-UTRs, ncRNAs, 5′-UTRs, nonessential coding regions (nCDS), essential coding regions (eCDS) or have no annotation (NOA). For example, state 1 HDEs often overlap essential coding regions, but S3 HDEs seldom do.

The HMM treatment of insertion data produced a data-driven partitioning of genomic elements based on the insertion model alone. To characterize the HMM-defined elements (HDEs) further, we compared their conservation during evolution and their RNA expression levels. The HDEs which were most insertion-depleted, and therefore most critical for cell function (S1 elements), covered 35% of the mappable genome. These HDEs showed distinct features: they were most conserved between species, the longest (mean length 1.9 kb), most highly expressed, and enriched for essential protein-coding regions (fig. 4). Another 52% of the genome was composed of S2 elements (mean length 1.0 kb), including mainly coding regions and UTRs, which also showed relatively high expression levels and conservation. The inclusion of many 5′- and 3′-UTRs in S1 and S2 elements indicates that these noncoding regions often contain regulatory sites whose disruption impairs cellular function. Finally, the S3 elements occupied only 3% of the genome, were seldom conserved, generally short (mean length 0.18 kb), were enriched for UTRs, ncRNAs, and unannotated regions. The UTRs likely contain regulatory sites, because insertion density is a predictor of constraint (see above). It would have been difficult to identify these regions without the insertion data because they are neither highly conserved nor highly transcribed. As the *Schizosaccharomyces* clade contains only four species, subtle constraint will likely remain undetected. In summary, HMM-defined regions were aligned to known annotation boundaries (fig. 3*A* and *B*), were consistent with evolutionary conservation and showed differences in transcription (fig. 4).

## Discussion

Dense transposon-insertion libraries can identify genes whose disruption affects fitness (in particular conditions) within bacterial genomes with high resolution (van Opijnen et al. 2009; Zhang et al. 2012; DeJesus and Ioerger 2013; Chao et al. 2016; Price et al. 2018). However, similarly high-resolution descriptions of eukaryotic genomes are more limited, and have not yet achieved nucleotide-level definitions of fitness landscapes (Guo et al. 2013; Michel et al. 2017). Studies with eukaryotic genomes are more challenging, because they are larger and contain nucleosomes, which bias integration rates. With the high density of insertions that we achieved (31 million insertions, 1 unique insertion site/13 nt), these data have potential to describe the functional significance of genomic segments at a very fine resolution.

As insertion positions are stochastic, we developed an HMM to define the discrete boundaries between insertion-depleted and insertion-rich regions. This approach demonstrated both strengths and weaknesses. Changes in HMM states were closely aligned to existing annotations (fig. 3*A*–*C*), and regions with continuous runs of one HMM state, identified elements with different properties (fig. 4), suggesting that the model partitioned genomic elements with different functions. The model was able to account for the known insertion biases: HMM states strongly depended on insertion density but only weakly correlated with nucleosome density and the insertion nucleotide motif (supplementary fig. 6, Supplementary Material online). Both raw insertion density and HMM model states could identify regions with enhanced evolutionary constraint, both genome-wide and within specific annotation categories, showing that the transposon data are broadly consistent with other fitness measures.

Other aspects of the HMM were less conclusive. While model fitting tests indicated that a five-state HMM was the best-supported, HMM states S4 and S5 were always present in short segments in the genome, and were not significantly different from each other in terms of evolutionary constraint. Moreover, mean HMM states for genes were only weakly correlated with gene knockout fitness (correlation coefficients ∼0.3). Either of these limitations may be due to the initial insertion data and/or the model. There could well be other insertion biases that are not accounted for, such as the position of a genomic segment in the 3D space of the nucleus (Michel et al. 2017). Such biases would limit our ability to predict the degree of genomic importance for regions that are refractive to transposon insertion. It is also possible that transposon insertions can disrupt the function of larger neighboring regions, although the sites of insertions themselves are not functional, which would inflate the HMM-based estimate of the functional genome. Finally, a limitation of any transposon insertion study is that the transposon method does not reveal how noncoding genomic elements function.

A simple model-free estimate, based on the assumption that 95% of essential coding regions are insertion-depleted, indicates that 66% of the genome contains functional elements. This is similar to the conclusion from comparative genomics that 68% from *Saccharomyces* is subject to evolutionary constraint (Siepel et al. 2005). Alternatively, based on the HMM, we would conclude that 91% of the fission yeast genome contains functional elements. In both cases, substantial proportions of the noncoding genomes appear to be insertion depleted (model-free 41%, HMM 80%). And in both cases, the insertion-depleted noncoding regions we define show statistically significant signals of enhanced constraint.

Comparative genomics is likely to produce conservative estimates of the functional proportions of genomes, because it is more likely to detect regions that have been continuously subject to purifying selection throughout the phylogeny of the species aligned (Stone et al. 2005). This will reduce our ability to detect regions that are subject to purifying selection in one species, but not another. As there are only four *Schizosaccharomyces* yeast genomes to align, we would expect a relatively ineffective detection of functional elements from comparative genomics in fission yeasts. Consistent with these caveats of functional genomics, we find that that 31% of the 100-nt windows of the genome are insertion depleted (mean HMM state ≤ 2), but have no signal of conservation between *Schizosaccharomyces* yeast genomes. The model-free estimate suggested that 20% of the genome is functional but has no signal of conservation. Both these analyses indicate that transposon mutagenesis can identify regions that are likely functional, but undetectable with the comparative genomics data available for this species.

Alternative analyses with different transposons, different species, or models will certainly be valuable. We expect that future work will reveal whether these elements function as the widespread noncoding transcripts (Atkinson et al. 2018) and/or as regulatory elements controlling the expression of coding genes.

## Conclusion

Our analysis indicates that the fission yeast genome is densely packed with functional elements, including many uncharacterized nonprotein-coding elements. Based on the HMM, we estimate that as much as 90% of the genome may contain functional elements that are impaired by transposon insertions, including between 40% and 80% of the nonprotein-coding regions. We conclude that saturating transposon mutagenesis data have potential to define functional nonprotein-coding elements within eukaryote genomes that would be difficult to detect with any other contemporary method.

## Materials and Methods

### Creating *Hermes* Insertion Libraries

*Hermes* insertion libraries were constructed as described (Park et al. 2009) using the pHL2577 and pHL2578 plasmids, except that the transposition frequency was calculated by dividing the number of colonies on YES 5-FOA+G418 plates by the number of colonies on YES plates. All experiments were performed in an *S. pombe* strain with the genotype *ura4*–D18 *leu1–32 h^–^*. Typically, <0.2% of cells in libraries contained genomic *Hermes* insertions, so we expect that most insertion mutants contain a single insertion.

### Generating DNA Libraries for Sequencing

Genomic DNA was extracted from insertion libraries using phenol/chloroform extraction. All DNA extracted from a library was processed. DNA was sheared to an average size of 200 bp using a Covaris S2 ultrasonicator (Covaris, Woburn, Massachusetts). Sheared DNA was end repaired using the NEBNext End Repair Module (NEB, Hitchin, UK). Linker1-Random10mer and Linker2 (supplementary table 4, Supplementary Material online) were ligated using the NEBNext Quick Ligation Module (NEB, Hitchin, UK). In Linker1-Random10mer, the random 10-nt sequence acted as a UMI to distinguish unique chromosomal insertions from PCR amplifications. DNA was then digested with KpnI-HF (NEB, Hitchin, UK) to exclude residual *Hermes* pHL2577 donor plasmid from PCR amplification (as the plasmid contains a unique KpnI site). NEBNext modules were used according to manufacturer’s instructions. To enrich for fragments containing the *Hermes* transposon, DNA was amplified with BIOTAQ DNA polymerase (Bioline, Essex, UK) using a primer complementary to the *Hermes* transposon (1-Transposon-4NNNN), and to the linker **(**Linker1-Amp, supplementary table 4, Supplementary Material online). Ultimately, a second PCR attached the multiplex oligonucleotides for Illumina MiSeq sequencing; the MS-102-2022 MiSeq reagent kit v2 (300 cycles) (Illumina, Cambridge, UK) was used to sequence the libraries. To increase the complexity of the libraries, for each library, ligation and PCR reactions were performed in multiple reactions (in 96-well plates), using a maximum of 1 µg of DNA per well and then repooled before sequencing. Detailed protocols are available in the Figshare project *Hermes Transposon Mutagenesis of the Fission Yeast Genome* (will be made publicly available upon manuscript acceptance). Sequence data are available at European Nucleotide Archive in study accession number PRJEB27324. Sample accessions are listed in supplementary table 5, Supplementary Material online.

### Computational Processing of Sequencing Data

Bioinformatic processing filtered the sequence data to retain only reads derived from *Hermes* insertions, removed reads with duplicate UMIs, and filtered for correctly paired high-confidence read-mapping, and ultimately located the positions and orientation (strand) of genomic insertions. Details are as follows. Read 1 architecture was [random4mer][*Hermes*][Genome] (with random 4mer added to increase 5′ Read 1 end complexity to allow Illumina cluster calling). The 4mer was trimmed with fastx_ trimmer (http://hannonlab.cshl.edu/fastx_toolkit/; Accessed on May 10, 2019). The Reaper tool (Davis et al. 2013) was used to detect reads with 5′ ends matching the expected *Hermes* sequence, and excluding those within the pHL2577 donor plasmid. Read 2 architecture was [10mer][Linker][Genome]. We used a custom Perl script to exclude duplicate reads with exactly matching 10mers. Processed Reads 1 and 2 were repaired using Tally (Davis et al. 2013), and the 10mer and Linker were trimmed with fastx_trimmer. Paired-end reads were aligned to the reference genome (Wood et al. 2002) and the donor plasmid using BWA-MEM (Li et al. 2009). SAMtools (Li et al. 2009) was used to select correctly paired reads with a mapping score ≥30 (flags 83 and 99). Finally, we applied custom scripts to identify the location and strand of insertions from the filtered BAM outputs with SAMtools. Insertions found on the same chromosome but on different strands were considered as unique events. Command lines for this procedure and scripts are available in the Figshare project *Hermes Transposon Mutagenesis of the Fission Yeast Genome*, as well as all insertion data, and HMM model fitting results.

### Nucleosome Density Data

The generation of the nucleosome density data has been described in Atkinson et al. (2018) and are available at the European Nucleotide Archive under accession number PRJEB21376. The median nucleosome density from two repeats was transformed to a normal distribution. This normalized nucleosome density showed a stronger correlation with insertion density than the raw nucleosome density and was used as a predictor in the HMM.

### Insertion Motif Similarity Score

In vitro *Hermes* insertion data (Guo et al. 2013) was used to identify a sequence motif corresponding to insertion events in nonnucleosome bound DNA. Strings of 41 nt, centered upon each in vitro insertion event were taken from the *S. pombe* reference sequence. The percentage of each nucleotide present at each of the 41 positions was measured and compared with percentage nucleotide compositions calculated across the entire genome. A window of 20 positions was identified for which the composition differed from the genome-wide composition by at least 1% for at least one of the four nucleotides. For each position *i*, we denote the probability of observing the nucleotide *a* as

$$p_{i}(a):1\leq i\leq 20,a\in \{A,G,C,T\}$$

and denote the genome-wide probability of observing the nucleotide *a* as p^gw^(a).

A genome-wide scan was then conducted of strings of 20 consecutive nt in the genome sequence, calculating a likelihood measure of the extent to which each string matched the insertion motif, as compared with the genome-wide base composition. Where a string is given by the nucleotides {*a*_1_, *a_2_*, …, *a*_20_} we calculate the insertion motif similarity score as follows:

$$\mathrm{IMSS}=\sum_{i=1}^{20}\left[\log p_{i}\left(a_{i}\right)-\log p^{\mathrm{gw}}\left(a_{i}\right)\right].$$

Here, a positive score indicates a greater similarity to the insertion motif than to the genome-wide sequence propensity. This likelihood measure was used as a predictor in the HMM.

### Hidden Markov Model

We developed an HMM using the R package depmixS4 (Visser and Speekenbrink 2010). These models assume that sequences of observed response variables are dependent on underlying sequences of discrete hidden states. The sequence of hidden states is assumed to follow a first-order Markov process, such that the probability of a state at position *t* depends only on the hidden state at the immediately preceding position *t*−1. The observed responses are assumed conditionally independent given the sequence of hidden states (i.e., correlations between nearby positions are completely accounted for by the hidden states). This model used log_2_-transformed insertion numbers as the observed state. Sites with zero insertions were set to observed state = 0. Each hidden state defined a (zero-inflated) Poisson regression model, with log_2_ insertion count as dependent variable, and the normalized nucleosome density (median of two replicates) and nucleotide preference score as predictors. Missing data for nucleosome density were set to the median. The models parameters (initial state probabilities, state-transition probabilities, and the parameters of the state-dependent zero-inflated Poisson regressions) were estimated by maximum likelihood using the Expectation–Maximization (EM) algorithm. Initial state distributions were all 1/*n*, where *n* is the number of states. Initial transition matrix was 0.95 for positions remaining in the same state, and 0.05/(*n*−1) for all other transitions. Initial parameter values of the Poisson regressions were obtained by pretraining each state-dependent model on a subset of the data (see below). These initial parameters were used to start the EM algorithm, the final resulting parameter estimates were determined by maximum likelihood. Neither annotations nor transcriptome data were supplied as predictors to the HMM. Models were fit to the insertion data by the EM algorithm, until convergence of the likelihood (with a tolerance 1×10^−8^) or with a maximum of 150 iterations (since log likelihood fit of models improved little after 150 iterations; supplementary fig. 7, Supplementary Material online).

### Choice of Optimal Model

To select an appropriate number of states and state training data for our HMM, we used ten “test data” subsets of the genome, each a 100-kb fraction as follows: Chromosome I, 100001–200001, 1100001–1200001, 2100001–2200001, 3100001–3200001, Chromosome II, 100001–200001, 1100001–1200001, 2100001–2200001, 3100001–3200001 and Chromosome III, 100001–200001, 1100001–1200001 (test data sets A to J). These regions avoid the chromosome ends, which have unusual properties, such as a high frequency of pseudogenes and native Tf1 transposon insertions (Jeffares et al. 2015).

We ran each of the following models on all insertion data from proliferating cells (split into the ten subsets). These models defined the training data in two ways. Firstly, “insertion-quantile” models, where training data were defined solely by the density of unique insertions, calculated over 100-nt windows. For example, a three-state model split the data into the lower, mid, and upper third insertion density for states 1–3. We trialed quantile models from two to ten states. Secondly, annotation-based models. We trialed 2-, 3-, 4-, and 5-state models where the training data were derived from current genome annotations. The two-state model included coding sequences (S1) and other regions (S2). The three-state model included coding sequences of essential genes (S1), coding sequences of nonessential genes (S2), introns, unannotated regions, and UTRs (S3). The four-state model included coding sequences of essential genes (S1), coding sequences of nonessential genes (S2), introns and untranslated regions (S3), and unannotated regions (S4). It differs from the three-state model in that it differentiates UTRs and introns from unannotated regions. The five-state model is as the four-state model, except that it includes a 5th state that contains sites with the highest 10% of unique insertions/100 nt. The response for this state was a Poisson distribution rather than zero-inflated Poisson.

Each of these 13 models was fit (with tolerance 1×10^−8^) to the ten fractions of the genome. Fitting involved optimizing the parameter of states at each position, the transition state matrix, and the slope, intercept, and zero-fraction of the state model. A five-state annotation model was chosen as a pragmatic best fit for running large (million position) data sets. Comparison of the Bayesian information criterion scores (BIC) for two to five states indicated that increasing states improved the fit (supplementary fig. 8, Supplementary Material online), but higher state models suffered from increased run times and frequent run failure, and/or highly inconsistent fractions of the subset data assigned to various states (with some states being absent).

Due to the rounding of log_2_ insertion counts, sites with one or zero insertions were set to the same observed state. Rounded log_2_ of insertions + 1 (where sites with zero insertions have different value from those with 1) resulted in a worse fit to the model (supplementary fig. 9, Supplementary Material online).

### Fitting of Chromosome-Wide Data

Once the five-state annotation model (model 5 A) was chosen as a pragmatic best model, it was run on all proliferation libraries, fitting data from five relatively equal portions of the genome separately, to allow runs in a practical time frame and memory. These fractions were: chromosome I left half (positions 1–2789566), chromosome I right half (positions 2789567–5579133), chromosome II left half (positions 1–2269902), chromosome II right half (positions 2269903–4539804), and the entirety of chromosome III (fractions are between 2.26 and 2.79 Mb). The model produced a state prediction for each position in the genome, and the posterior probability of each state at each position.

These separate fits to the model resulted in similar distributions of states between chromosome arms for both the coding regions and introns of essential genes, supporting consistent convergence of the models between these genome subsets (supplementary fig. 10, Supplementary Material online). To examine whether positions were assigned a consistent state using different subsets of data, and independent fits of the HMM, we made subsets of proliferation (dense data) for the central half of chromosome I (positions 1394783–4184350), which overlaps both the left and right halves used previously. These data were fit to model 5 A as before. With dense proliferation data, sites that overlapped the 96.7% of positions were assigned the same state with either left versus middle, or right versus middle comparisons. States 1–5 were all consistently assigned (e.g., > 99% of state five positions were the same within proliferation data, and similar proportions for all other states). This analysis indicates that these fractions were sufficiently large to preclude fitting to very different local optima. HMM code is available in the Figshare project *Hermes Transposon Mutagenesis of the Fission Yeast Genome*.

### Filtering Badly Mapped Sites

To ensure accurate placement of reads, our pipeline filtered reads mapped with mapping quality ≥30. To avoid the tendency to misinterpret regions that have few insertions due to the loss of low mapping quality, we analyzed only sites that had retained ≥90% of the reads (lost <10%) over 500-nt windows after mapping quality filtering. This retained 94.6% of the genome for analysis. After filtering, there was only a weak negative correlation between the HMM state and the proportion of reads filtered (Pearson *r* = −0.049). All data presented included only the sites that had retained ≥90% of the reads after filtering for Q30 mapping (the “mappable genome”).

### Annotation Data

Annotations were from PomBase (ASM294v2, February 11, 2016), including 1,538 annotated ncRNAs.

### Transcriptome Analysis

Replicated RNA-Seq data from vegetatively growing, early stationary, and deep stationary cultures were retrieved from the European Nucleotide Archive (ENA; http://www.ebi.ac.uk/ena; Accessed on May 10, 2019) using the following accession numbers (data set: PRJEB7403; samples: ERS555567, ERS555607, ERS555570, ERS555612, ERS555571, ERS555613) (Atkinson et al. 2018). Reads were aligned to the *S. pombe* genome as described (Bitton et al. 2014). The resultant aligned reads were used to compute normalized coverage at the nucleotide level using the genomecov function in the BEDtools suite (Quinlan and Hall 2010). Customized R scripts were used to define whether a given region is transcribed.

### Comparative Genomics

We used updated genome assemblies of fission yeasts *S. octosporus*, *S. japonicus*, and *S. cryophilus* (Tong et al. 2018). To improve previous full genome alignments of fission yeast species (Rhind et al. 2011), we incorporated these newly assembled genomes into an alignment with the S*. pombe* genome using progressive-cactus (Paten et al. 2011) (github version May 2016), using a guide tree based on Rhind et al. (2011). We then applied the phyloP algorithm (Siepel et al. 2006) as implemented in the HAL toolkit (Hickey et al. 2013) to detect constraints. We trained a neutral model using the 4-fold degenerate sites from coding regions from the high-quality *S. pombe* annotation.

### 100-Nucleotide Window Analysis

Analysis of 100-nt windows used custom scripts to calculate mean HMM state, unique insertions/nt, and mean phyloP signal. Annotation analysis for 100-nt windows used windows where 100% of the window was covered by the annotation in question.

## Supplementary Material

Supplementary data are available at *Molecular Biology and Evolution* online.

## Supplementary Material

msz113_Supplementary_Data

## Notes

Sequence data are available at European Nucleotide Archive in study accession number PRJEB27324. Sample accessions are listed in supplementary table 5, Supplementary Material online. Transposon insertion data, R code for the HMM, all other data used for analysis, and detailed protocols are available in the figshare project *Hermes Transposon Mutagenesis of the Fission Yeast Genome*.

ORCiDs: PHS: 0000-0001-6776-0934, DCJ: 0000-0001-7320-0706, JB: 0000-0003-4036-1532, CJRI: 0000-0002-0030-2784, VAT: 0000-0001-9526-5957, CYS: 0000-0001-7416-1470

## References

[msz113-B1] Atkinson SR, Marguerat S, Bitton DA, Rodríguez-López M, Rallis C, Lemay J-F, Cotobal C, Malecki M, Smialowski P, Mata J, et al 2018 Long noncoding RNA repertoire and targeting by nuclear exosome, cytoplasmic exonuclease, and RNAi in fission yeast. RNA 24 9:1195–1213. 29914874 10.1261/rna.065524.118 PMC6097657

[msz113-B2] Bitton DA, Rallis C, Jeffares DC, Smith GC, Chen YY, Codlin S, Marguerat S, Bähler J. 2014 LaSSO, a strategy for genome-wide mapping of intronic lariats and branch-points using RNA-seq. Genome Res. 24 7:1169–1179. 24709818 10.1101/gr.166819.113 PMC4079972

[msz113-B3] Campos JL, Halligan DL, Haddrill PR, Charlesworth B. 2014 The relation between recombination rate and patterns of molecular evolution and variation in *Drosophila melanogaster*. Mol Biol Evol. 31 4:1010–1028. 24489114 10.1093/molbev/msu056 PMC3969569

[msz113-B4] Chao MC, Abel S, Davis BM, Waldor MK. 2016 The design and analysis of transposon insertion sequencing experiments. Nat Rev Microbiol. 14 2:119–128. 26775926 10.1038/nrmicro.2015.7 PMC5099075

[msz113-B5] Cheeseman IH, Miller BA, Nair S, Nkhoma S, Tan A, Tan JC, Saai Al S, Phyo AP, Moo CL, Lwin KM, et al 2012 A major genome region underlying artemisinin resistance in malaria. Science 336 6077:79–82. 22491853 10.1126/science.1215966 PMC3355473

[msz113-B6] Davis MPA, Van Dongen S, Abreu-Goodger C, Bartonicek N, Enright AJ. 2013 Kraken: a set of tools for quality control and analysis of high-throughput sequence data. Methods 63 1:41–49. 23816787 10.1016/j.ymeth.2013.06.027 PMC3991327

[msz113-B7] DeJesus MA, Ioerger TR. 2013 A hidden Markov model for identifying essential and growth-defect regions in bacterial genomes from transposon insertion sequencing data. BMC Bioinformatics 14:303. 24103077 10.1186/1471-2105-14-303 PMC3854130

[msz113-B8] Doolittle WF. 2013 Is junk DNA bunk? A critique of ENCODE. Proc Natl Acad Sci U S A. 110 14:5294–5300. 23479647 10.1073/pnas.1221376110 PMC3619371

[msz113-B9] ENCODE Project Consortium, Bernstein BE, Birney E, Dunham I, Green ED, Gunter C, Snyder M. 2012 An integrated encyclopedia of DNA elements in the human genome. Nature 489:57–74. 22955616 10.1038/nature11247 PMC3439153

[msz113-B10] Evertts AG, Plymire C, Craig NL, Levin HL. 2007 The hermes transposon of *Musca domestica* is an efficient tool for the mutagenesis of *Schizosaccharomyces pombe*. Genetics 177 4:2519–2523. 17947404 10.1534/genetics.107.081075 PMC2219505

[msz113-B11] Fawcett JA, Iida T, Takuno S, Sugino RP, Kado T, Kugou K, Mura S, Kobayashi T, Ohta K, Nakayama J-I, et al 2014 Population genomics of the fission yeast *Schizosaccharomyces pombe*. PLoS One 9 8:e104241. 25111393 10.1371/journal.pone.0104241 PMC4128662

[msz113-B12] Gagliano SA, Barnes MR, Weale ME, Knight J. 2014 A Bayesian method to incorporate hundreds of functional characteristics with association evidence to improve variant prioritization. PLoS One 9 5:e98122. 24844982 10.1371/journal.pone.0098122 PMC4028284

[msz113-B13] Gangadharan S, Mularoni L, Fain-Thornton J, Wheelan SJ, Craig NL. 2010 Inaugural Article: DNA transposon Hermes inserts into DNA in nucleosome-free regions in vivo. Proc Natl Acad Sci U S A. 107 51:21966–21972. 21131571 10.1073/pnas.1016382107 PMC3009821

[msz113-B14] Graur D, Zheng Y, Price N, Azevedo RBR, Zufall RA, Elhaik E. 2013 On the immortality of television sets: “function” in the human genome according to the evolution-free gospel of encode. Genome Biol Evol. 5 3:578–590. 23431001 10.1093/gbe/evt028 PMC3622293

[msz113-B15] Guo Y, Park JM, Cui B, Humes E, Gangadharan S, Hung S, Fitzgerald PC, Hoe K-L, Grewal SIS, Craig NL, et al 2013 Integration profiling of gene function with dense maps of transposon integration. Genetics 195 2:599–609. 23893486 10.1534/genetics.113.152744 PMC3781984

[msz113-B16] Hickey G, Paten B, Earl D, Zerbino D, Haussler D. 2013 HAL: a hierarchical format for storing and analyzing multiple genome alignments. Bioinformatics 29 10:1341–1342. 23505295 10.1093/bioinformatics/btt128 PMC3654707

[msz113-B17] Jeffares DC, Rallis C, Rieux A, Speed D, Převorovský M, Mourier T, Marsellach FX, Iqbal Z, Lau W, Cheng TMK, et al 2015 The genomic and phenotypic diversity of *Schizosaccharomyces pombe*. Nat Genet. 47 3:235–241. 25665008 10.1038/ng.3215 PMC4645456

[msz113-B19] Kim D-U, Hayles J, Kim D, Wood V, Park H-O, Won M, Yoo H-S, Duhig T, Nam M, Palmer G, et al 2010 Analysis of a genome-wide set of gene deletions in the fission yeast *Schizosaccharomyces pombe*. Nat Biotechnol. 28 6:617–623. 20473289 10.1038/nbt.1628 PMC3962850

[msz113-B20] Li H, Handsaker B, Wysoker A, Fennell T, Ruan J, Homer N, Marth G, Abecasis G, Durbin R, 1000 Genome Project Data Processing Subgroup. 2009 The Sequence Alignment/Map format and SAMtools. Bioinformatics 25 16:2078–2079. 19505943 10.1093/bioinformatics/btp352 PMC2723002

[msz113-B23] Malecki M, Bähler J. 2016 Identifying genes required for respiratory growth of fission yeast. Wellcome Open Res. 1:12. 27918601 10.12688/wellcomeopenres.9992.1 PMC5133385

[msz113-B24] Malecki M, Bitton DA, Rodríguez-López M, Rallis C, Calavia NG, Smith GC, Bähler J. 2016 Functional and regulatory profiling of energy metabolism in fission yeast. Genome Biol. 17 1:240. 27887640 10.1186/s13059-016-1101-2 PMC5124322

[msz113-B25] Michel AH, Hatakeyama R, Kimmig P, Arter M, Peter M, Matos J, De Virgilio C, Kornmann B. 2017 Functional mapping of yeast genomes by saturated transposition. eLife 6:E3179. 10.7554/eLife.23570 PMC5466422 28481201

[msz113-B26] Park JM, Evertts AG, Levin HL. 2009 The Hermes transposon of *Musca domestica* and its use as a mutagen of *Schizosaccharomyces pombe*. Methods 49 3:243–247. 19450689 10.1016/j.ymeth.2009.05.004 PMC2782614

[msz113-B27] Paten B, Earl D, Nguyen N, Diekhans M, Zerbino D, Haussler D. 2011 Cactus: algorithms for genome multiple sequence alignment. Genome Res. 21 9:1512–1528. 21665927 10.1101/gr.123356.111 PMC3166836

[msz113-B28] Price MN, Wetmore KM, Waters RJ, Callaghan M, Ray J, Liu H, Kuehl JV, Melnyk RA, Lamson JS, Suh Y, et al 2018 Mutant phenotypes for thousands of bacterial genes of unknown function. Nature 44:D330–D509. 10.1038/s41586-018-0124-0 29769716

[msz113-B29] Quinlan AR, Hall IM. 2010 BEDTools: a flexible suite of utilities for comparing genomic features. Bioinformatics 26 6:841–842. 20110278 10.1093/bioinformatics/btq033 PMC2832824

[msz113-B31] Rhind N, Chen Z, Yassour M, Thompson DA, Haas BJ, Habib N, Wapinski I, Roy S, Lin MF, Heiman DI, et al 2011 Comparative functional genomics of the fission yeasts. Science 332 6032:930–936. 21511999 10.1126/science.1203357 PMC3131103

[msz113-B32] Sideri T, Rallis C, Bitton DA, Lages BM, Suo F, Rodríguez-López M, Du L-L, Bähler J. 2014 Parallel profiling of fission yeast deletion mutants for proliferation and for lifespan during long-term quiescence. G3 (Bethesda) 5 1:145–155. 25452419 10.1534/g3.114.014415 PMC4291465

[msz113-B33] Siepel A, Bejerano G, Pedersen JS, Hinrichs AS, Hou M, Rosenbloom K, Clawson H, Spieth J, Hillier LW, Richards S, et al 2005 Evolutionarily conserved elements in vertebrate, insect, worm, and yeast genomes. Genome Res. 15 8:1034–1050. 16024819 10.1101/gr.3715005 PMC1182216

[msz113-B34] Siepel A, Pollard KS, Haussler D. 2006 New methods for detecting lineage-specific selection In: Research in computational molecular biology. Vol. 3909. Lecture notes in computer science. Heidelberg (Berlin): Springer Berlin Heidelberg p. 190–205.

[msz113-B35] Smith JM, Haigh J. 1974 The hitch-hiking effect of a favourable gene. Genet Res. 23 1:23–35. 4407212

[msz113-B36] Stone EA, Cooper GM, Sidow A. 2005 Trade-offs in detecting evolutionarily constrained sequence by comparative genomics. Annu Rev Genomics Hum Genet. 6:143–164. 16124857 10.1146/annurev.genom.6.080604.162146

[msz113-B37] Tong P, Pidoux AL, Toda NR, Ard R, Berger H, Shukla M, Torres-Garcia J, Mueller CA, Nieduszynski CA, Allshire RC. 2018 Inter-species conservation of organisation and function between non-homologous regional centromeres. bioRxiv 309815. 10.1038/s41467-019-09824-4 PMC6538654 31138803

[msz113-B38] van Opijnen T, Bodi KL, Camilli A. 2009 Tn-seq: high-throughput parallel sequencing for fitness and genetic interaction studies in microorganisms. Nat Methods. 6 10:767–772. 19767758 10.1038/nmeth.1377 PMC2957483

[msz113-B39] Visser I, Speekenbrink M. 2010 depmixS4: An R-package for hidden Markov models. *J Stat Softw*.

[msz113-B41] Wood V, Gwilliam R, Rajandream M-A, Lyne M, Lyne R, Stewart A, Sgouros J, Peat N, Hayles J, Baker S, et al 2002 The genome sequence of *Schizosaccharomyces pombe*. Nature 415 6874:871–880. 11859360 10.1038/nature724

[msz113-B42] Yu F, Lu J, Liu X, Gazave E, Chang D, Raj S, Hunter-Zinck H, Blekhman R, Arbiza L, Van Hout C, et al 2015 Population genomic analysis of 962 whole genome sequences of humans reveals natural selection in non-coding regions. *PLoS One* 10:e0121644. 10.1371/journal.pone.0121644 PMC4373932 25807536

[msz113-B43] Zhang YJ, Ioerger TR, Huttenhower C, Long JE, Sassetti CM, Sacchettini JC, Rubin EJ. 2012 Global assessment of genomic regions required for growth in *Mycobacterium tuberculosis*. PLoS Pathog. 8 9:e1002946. 23028335 10.1371/journal.ppat.1002946 PMC3460630

