# Supplementary material for: Fitness Landscape of the Fission Yeast Genome
Source: Mol Biol Evol. 2019 May 11;36(8):1612–23. doi: 10.1093/molbev/msz113 (PMC6657727; doi:10.1093/molbev/msz113)
Supplement: msz113_Supplementary_Data [file msz113_supplementary_data.zip › MBE-18-0848-R3-supplementary-figs.docx]

**Fitness Landscape of the Fission Yeast Genome**

Leanne Grech^1*^, Daniel C. Jeffares^1,2*†^, Christoph Y. Sadée^1^, María Rodríguez-López^1^, Danny A. Bitton^1^, Mimoza Hoti^1^, Carolina Biagosch^1^, Dimitra Aravani^1^, Maarten Speekenbrink^4^, Christopher J. R. Illingworth^5^, Philipp H. Schiffer^1^, Alison L. Pidoux^6^, Pin Tong^6^, Victor A. Tallada^3^, Robin Allshire^6^, Henry L. Levin^7^ & Jürg Bähler^1,8†^.

**Contributed Equally*.

†*Corresponding Authors*: [daniel.jeffares@york.ac.uk](mailto:daniel.jeffares@york.ac.uk), [j.bahler@ucl.ac.uk](mailto:j.bahler@ucl.ac.uk).

ORCiDs: PHS: 0000-0001-6776-0934, DCJ: 0000-0001-7320-0706, JB: 0000-0003-4036-1532, CJRI: 0000-0002-0030-2784, VAT: 0000-0001-9526-5957, CYS: 0000-0001-7416-1470

Affiliations:

1. Department of Genetics, Evolution and Environment, Gower Street – Darwin Building, University College London, London, WC1E 6BT, UK.
2. Department of Biology, University of York, Wentworth Way, York, YO10 5DD, UK.
3. Centro Andaluz de Biología del Desarrollo, Universidad Pablo de Olavide/Consejo Superior de Investigaciones Científicas, Carretera de Utrera Km1, 41013, Seville, Spain.
4. Experimental Psychology, University College London, 26 Bedford Way, London, WC1H 0AP, UK.
5. Department of Genetics, University of Cambridge, Downing Street, Cambridge, CB2 1EH, UK.
6. Wellcome Trust Centre for Cell Biology, University of Edinburgh, Michael Swann Building, Max Born Crescent, Edinburgh, EH9 3BF, UK.
7. Division of Molecular and Cellular Biology, Eunice Kennedy Shriver National Institute of Child Health and Human Development, National Institutes of Health, Bethesda, MD 20892, USA.
8. UCL Genetics Institute, University College London, London, WC1E 6BT, UK.

**Supplementary Figures**

**Supplementary fig. 1. Percentage of cells with a chromosomal insertion.**

For the nine libraries we generated (and others not described here), we show the percentage of cells with a chromosomal insertion. The proportion was calculated as the number of colonies present on YES + FOA + G418 plates (chromosomal insertions), divided by the number of colonies present on YES plates (all cells).

**Supplementary fig. 2. The custom *Hermes*-end primed sequencing strategy.** Shows the end-priming strategy used to sequence Hermes-containing fragments. Initially, genomic DNA is extracted, sheared, end repaired, and linkers (Linker1-Random10mer and Linker2) ligated at both terminal ends (1). To enrich for fragments containing the *Hermes* transposon, DNA was amplified with using a primer that is complimentary to the *Hermes* transposon (1-Transposon-4NNNN) (2), and to the linker **(**Linker1-Amp) (3), to produce fragments that contain linkers, genomic DNA and the *Hermes* right terminal inverted repeat (4). A second PCR attached the multiplex oligonucleotides for Illumina sequencing (5,6), producing the final product that is sequenced (7). Detailed protocols are available in the Figshare project *Hermes Transposon Mutagenesis of the Fission Yeast Genome*.

**Supplementary fig. 3. Properties of insertions in different annotation regions.**

Left panel shows average insertion count in coding regions of essential genes, pseudogenes, other (non-essential) coding regions, introns, canonical non-coding RNAs (snoRNas, tRNAs, rRNAs, snRNAs), long terminal repeats of transposons, 5’ and 3’ untranslated regions, regions with no annotation and intergenic long non-coding RNAs. Middle panel shows average insertion count (all sites, including sites with no insertions) for the same annotation classes. Right panel shows average insertion density (unique insertion positions/site) for the same annotations.

**Supplementary fig.** **4. Insertions in the mitochondrial genome.**

Unique insertions per site in the mitochondrial genome showed little difference between coding and non-coding regions, whereas the nuclear genome showed far fewer insertions in the coding regions.

**Supplementary fig. 5. Relationships between insertion density, nucleosome density and the insertion motif similarity score.**

All plots show relationships with mean insertion count for sites with Hermes insertions (left panels) or mean insertions/site. In each case, the genome was divided into 100 partitions according to the measure on the *x* axis, and the insertion counts or insertion densities were calculated from these partitions. A) insertion counts plotted against normalised nucleosome density (nucsome.norm). B) insertion density plotted against normalised nucleosome density. C) log scale insertion counts plotted against log scale normalised nucleosome density. D) log scale insertion density plotted against log scale normalised nucleosome density. E) insertion counts plotted against insertion motif similarity score (IMSS). F) insertion density plotted against insertion motif similarity score.

**Supplementary fig. 6. HMM states strongly depended on insertion density but only weakly correlated with nucleosome density and nucleotide motif.**

Top row; for coding regions we show the relationship between HMM states defined and insertion density (unique insertions/100 nt) (left panel), normalised nucleosome density (nsome.norm, middle panel) and the insertion motif similarity score (nt.model, right panel). Middle row; the same relationships for 5’ and 3’ untranslated regions. Lower row, the same relationships for regions with no annotations. In all cases Spearman rank correlations are shown above plots.

**Supplementary fig. 7. Log likelihoods for fits of HMM models improved little after 150 iterations.** For sections of chromosomes I, II and III we show the log likelihood of the model fit to the data with successive iterations of the Viterbi algorithm. Left panels show the entire range of likelihoods, with red and green dashed lines showing the 95^th^ and 99^th^ percentiles. Right panels show the upper 5^th^ percentiles. Model fits improved little after 150 iterations.

**Supplementary fig. 8. Bayesian information criterion scores (BIC) indicated that the 5-state annotation model was the best fit.** For ten 100 kb fractions of the genome (data sets A – J), we show the BIC scores for model fitting with the depmixS4 package (Visser and Speekenbrink 2010; Visser and Speekenbrink 2015). Red points show the annotation-based models from 2-5 states (see methods for state definitions). Black points show the quantile models, where training data is defined based on insertion density quantiles (unique insertions/100 nt). For example a three-state model used the first third of insertion-dense data to train S1, the second third to train S2, *etc*. The five-state model which was used for this analysis was trained on coding sequences of essential genes (S1), coding sequences of non-essential genes (S2), introns and untranslated regions (S3), and unannotated regions (S4), and sites with the highest 10% of unique insertions/100 nt (S5). The ten ‘test data’ subsets of the genome, each a 100 kb fraction as are follows: Chromosome I, 100001-200001, 1100001-1200001, 2100001-2200001, 3100001-3200001, Chromosome II, 100001-200001, 1100001-1200001, 2100001-2200001, 3100001-3200001 and Chromosome III, 100001-200001, 1100001-1200001 (test data sets A to J).

**Supplementary fig. 9. Excluding singleton insertions produced better model fits.**

HMM code used log_2_ of insertion counts (rounded to the nearest integer). Since log_2_(1) is zero, this treats sites with one insertion the same as sites with no insertions. Trails of the HMM code that used log_2_(insertions+1), where sites with 0 insertions have different value from those with 1, resulted in a worse fit to the model. For two of the test data sets (A, J), we show the BIC for models fitted with log_2_(insertions) and log_2_(insertions+1).

**Supplementary fig. 10. Separate fits to the model with different data resulted in similar distributions of states.** Model fitting was performed on five subsets of the data; IL (left arm of chromosome I), IR (right arm of chromosome I), IIL (left arm of chromosome II), IIR (right arm of chromosome II), and III (all of chromosome III). The left panel shows the proportion of essential coding regions for each subset that were assigned to states 1-5, according to the key. Most were assigned to state 1 or 2. The right panel shows the –log10 of the proportion, which indicates that the less frequent states are also similarly distributed between subset model fits, supporting consistent convergence of the model between these genome subsets.

**References**

Visser I, Speekenbrink M. 2010. depmixS4: An R-package for hidden Markov models. Journal of Statistical Software.

Visser I, Speekenbrink M. 2015. depmixS4: Dependent Mixture Models - Hidden Markov Models of GLMs and Other Distributions in S4. (Version 1.3-3). [Software]. (2015).
